# Diagnosis of Fibrotic Hypersensitivity Pneumonitis: Is There a Role for Biomarkers?

**DOI:** 10.3390/life13020565

**Published:** 2023-02-17

**Authors:** João O. Pereira, Vânia Fernandes, Tiago M. Alfaro, Sara Freitas, Carlos Robalo Cordeiro

**Affiliations:** 1 Department of Pulmonology, Centro Hospitalar e Universitário de Coimbra, 3000-561 Coimbra, Portugal; 2 Faculty of Medicine, University of Coimbra, 3000-504 Coimbra, Portugal; 3 Department of Pulmonology, Centro Hospitalar do Baixo Vouga, 3810-501 Aveiro, Portugal

**Keywords:** hypersensitivity pneumonitis, extrinsic allergic alveolitis, pulmonary fibrosis, biomarkers, early diagnosis

## Abstract

Hypersensitivity pneumonitis is a complex interstitial lung syndrome and is associated with significant morbimortality, particularly for fibrotic disease. This condition is characterized by sensitization to a specific antigen, whose early identification is associated with improved outcomes. Biomarkers measure objectively biologic processes and may support clinical decisions. These tools evolved to play a crucial role in the diagnosis and management of a wide range of human diseases. This is not the case, however, with hypersensitivity pneumonitis, where there is still great room for research in the path to find consensual diagnostic biomarkers. Gaps in the current evidence include lack of validation, validation against healthy controls alone, small sampling and heterogeneity in diagnostic and classification criteria. Furthermore, discriminatory accuracy is currently limited by overlapping mechanisms of inflammation, damage and fibrogenesis between ILDs. Still, biomarkers such as BAL lymphocyte counts and specific serum IgGs made their way into clinical guidelines, while others including KL-6, SP-D, YKL-40 and apolipoproteins have shown promising results in leading centers and have potential to translate into daily practice. As research proceeds, it is expected that the emergence of novel categories of biomarkers will offer new and thriving tools that could complement those currently available.

## 1. Introduction

Hypersensitivity pneumonitis (HP) is a complex interstitial lung syndrome characterized by an immunological reaction to a wide variety of organic or low-molecular-weight chemical particles [1]. This immunological reaction arises with repeated and prolonged inhalation of an environmental antigen, to which the individual is sensitized [2].

Early findings suggest the disease pathogenesis is mediated by circulating specific IgG antibodies (precipitins) through the formation of antigen–antibody complexes, in a type III hypersensitivity reaction [3,4,5]. This hypothesis was challenged by the absence of precipitins in some patients, the absence of vasculitis and neutrophilic infiltrates in tissue samples and the lack of histological features of HP in animal models injected with precipitins and exposed to inciting antigens [6,7]. The important role of innate immune response was later described and both mechanisms of type III and type IV hypersensitivity reactions are currently thought to occur [8]. Polymorphisms of genes encoding molecules involved in antigen processing and presentation, remarkably those located on the major histocompatibility complex II region, appear to increase the susceptibility to a pathological sensitization process. Furthermore, the T-cell effector subset response appears to influence histological patterns, with skewing toward Th2 activity correlating with sustained inflammation and fibrotic response [8,9,10,11]. In addition to the inciting antigens, exposure to other external agents such as bacteria, fungi, mites and other inert particles and chemicals is thought to contribute to HP pathogenesis [8].

Despite efforts by international groups and scientific societies, a wide consensus on disease definition is still missing and international clinical practice guidelines on the diagnosis were only recently published for the first time [12,13]. HP was classically categorized as acute, subacute or chronic, depending on the pattern of exposure and duration of illness [14], but patients followed heterogeneous clinical courses irrespective of this category [15]. The need to add prognostic value led to a recent classification of the disease into fibrotic and non-fibrotic, according to clinical, pathological and/or imaging characteristics [12,13,16]. However, heterogeneity in clinical presentation, type and duration of exposure, cultural practices, geographical conditions and host risk factors still contribute for the under-recognition and underdiagnosis of HP. Furthermore, significant overlap of features with other clinical entities helps explain the disappointing agreement levels between multidisciplinary teams on the diagnosis (kappa value 0.29 [0.24–0.40]) [17] and common misdiagnosis with idiopathic pulmonary fibrosis (IPF) and other idiopathic interstitial pneumonias [18,19]. The variety of antigens associated with the disease, currently estimated to be more than 200 [20] and the lack of standardized techniques for testing for hypersensitivity pose as additional obstacles, as the inability to identify the relevant exposure may hamper the diagnosis and is associated with worse outcomes [21].

Altogether, these factors may partially explain discrepancies in HP frequency among different world regions and even within the same country [22,23,24,25]. Even though overall HP frequency appears to be low [26,27,28,29,30], a different perspective might be gained from cohorts and registries of interstitial lung disease (ILD), which report that HP may account for up to 47.3% of incident ILD cases [23,31]. Limited evidence details the epidemiology of acute vs. chronic HP, but approximately 40–50% of the latter corresponds to fibrotic disease [32], whose poor prognosis should be noted. With 7-year survival rates recently estimated in 40.8% [33], fibrotic HP presents poorer outcomes than many cancers [34]. The HP-associated mortality rate appears to be increasing [35], further highlighting the urge for improving accuracy and time to diagnosis and exposure identification, as these are crucial for improving the outcomes of the disease.

## 2. Biomarkers in Fibrotic HP

Biomarkers can be defined as “characteristics that are objectively measured and evaluated as an indicator of normal biologic processes, pathogenic processes or pharmacologic responses to a therapeutic intervention” [36]. They can be classified according to their putative application as (a) diagnostic, (b) monitoring, (c) pharmacodynamic/response, (d) predictive, (e) prognostic, (f) safety and (g) susceptibility/risk. Even though different subtypes may overlap, it is essential to pursue evidence and validation for each particular use [37]. This is the step where the majority of biomarkers fail before reaching clinical practice [38]. 

Even though the application of biomarkers has become routine in research and has potential to become central in clinical practice, their adoption strongly depends on their accuracy for each putative use and population, reproducibility, affordability, availability and invasiveness [39].

The development of a biomarker can be made using system biology methods to screen a large quantity of potential biomarkers for their association with the disease (unbiased approach) or selecting individual candidate biomarkers according to pre-existing knowledge on the mechanisms of disease. [39].

Sampling the alveolar milieu with bronchoalveolar lavage (BAL) may be indicated in ILDs, not only to study microbiological agents and cell populations [40], but also cell markers, soluble mediators (e.g., proteins secreted by the epithelium). On the other hand, significant deviations in the serum levels of some molecules may occur due to local overproduction and increased permeability and/or destruction of the air-blood barrier in the diseased lung [41,42,43]. This led to their potential use as serum biomarkers with diverse purposes [44]. 

This article aims to review the literature on biomarkers that can be quantified in the serum and/or BAL with the potential to contribute to the diagnosis of HP by discriminating patients with fibrotic HP from individuals without documented ILD or those with other ILDs. For brevity, only molecular (excluding genomic) and differential cell biomarkers are reviewed and classified according to their proposed pathobiological mechanisms of origin [45,46,47,48]. 

We searched MEDLINE using the query “hypersensitivity pneumonitis AND biomarker”. Clinical and preclinical original articles, reviews and statement papers potentially addressing the topic were selected for full-text analysis and included in our review after deemed relevant. This was complemented by a secondary analysis of the references of the manuscripts whose full texts were examined and by a MEDLINE search on specific biomarkers using the query “hypersensitivity pneumonitis AND (specific biomarker)”.

### 2.1. Immune Dysregulation

As mentioned, the pathogenesis of HP involves a complex, dysregulated cascade of immunological events in predisposed individuals. In the airway, inciting molecules are firstly recognized by pattern-recognition receptors, phagocyted, processed and expressed at the surface of innate immune cells, starting the sensitization process. This is followed by activation of adaptive immune cells and the production of several proteins, including antibodies, cytokines and chemokines [8]. The resulting inflammation leads to the apoptosis of alveolar epithelial cells, which may itself contribute to further recruitment of immune cells [49]. The differential expression of these molecules and cell subtypes has been explored as a potential diagnostic tool in HP and is summarized in Table 1. 

#### 2.1.1. BAL Lymphocyte Count

BAL lymphocytosis reflects the degree of alveolitis in chronic HP, a major characteristic of this disease [50]. These counts appear to be less pronounced in the presence of fibrosis [51,52,53,54], but their relationship with antigen exposure, host factors and disease progression is incompletely understood.

In fact, though BAL lymphocyte counts have been proposed as a diagnostic criterion for HP [55,56,57], robust evidence clarifying their role in diagnosing chronic HP [15] and their additive discriminative value in differentiating it from other fibrotic ILDs remains altogether little determined [58].

Tzilas et al. reported an added diagnostic yield for BAL lymphocytosis (using a cutoff of >20%) in a retrospective cohort of ILDs with an indeterminate for UIP pattern on high-resolution computerized tomography. In their study, BAL lymphocytosis led to a change in the diagnosis to HP in 14.7% of the patients [59].

A meta-analysis including 49 case series found significantly higher BAL lymphocyte percentages in HP and in the subgroup of fibrotic HP when compared with IPF and with sarcoidosis. However, threshold analysis for fibrotic HP vs. IPF and fibrotic HP vs. sarcoidosis yielded disappointing discriminative ability (area under curve (AUC) 0.54 (95% CI 0.51–0.58) and 0.44 (95% CI 0.41–0.47), respectively) [60].

Another systematic review (53 studies) and meta-analysis (42 studies) by Adderley et al. found a pooled estimate for the BAL lymphocyte percentage in chronic HP (cHP) of 42.8%, significantly higher than in IPF and non-IPF idiopathic interstitial pneumonias. However, the authors did not find significant differences in the BAL lymphocyte count from CTD-ILDs and sarcoidosis [61]. 

Inference from the existing evidence may be significantly limited by aggregation of studies with heterogeneous diagnostic criteria and incorporation bias. Still, the role of BAL lymphocyte counts in distinguishing fibrotic HP from IPF and sarcoidosis is currently recognized [12]. However, due to severe heterogeneity and limited number of studies, ideal cutoff values cannot be established, nor their role in differentiating chronic HP from other ILDs.

To conclude, a high BAL lymphocyte count is likely to be more useful for establishing diagnostic likelihood of HP in the setting of multidisciplinary discussion, in conjunction with other diagnostic elements, rather than relied upon its own [12,13].

#### 2.1.2. BAL CD4/CD8 Ratio

CD4 lymphocyte predominance and increased CD4/CD8 ratios on the BAL have been documented in chronic HP, when compared with acute and subacute forms but the use of these markers to diagnose or exclude the disease in the clinical setting is limited by inconsistent findings and significant variation with external factors [9,62,63,64,65,66,67,68,69,70]. Moreover, CD4/CD8 ratio analysis does not appear to improve diagnostic accuracy in ILD when added to cell count analysis [71].

Still, in spite of significant between-study variability, pooled estimates point to lower CD4/CD8 ratios in HP when compared with sarcoidosis [61] and a high ratio (>3.5–4) is currently considered highly specific and useful to support the latter [72,73]. The authors, thus, consider that this parameter may be valuable in specific settings where the differential diagnosis of these two conditions may be challenging.

#### 2.1.3. Cytokines and Cytokine Receptors

Upon exposure to the inciting antigen, antigen presenting cells (APCs) release a panoply of cytokines including IFN-γ, IL-1, IL-6, IL-12, IL-18 and TNF-α, and there appears to be no difference in the BAL concentrations of most of these molecules between patients with acute and chronic HP [74]. Meanwhile, upon being presented with processed antigens, T-cell response can evolve to a variety of subsets. While Th1 milieu (e.g., IFN-γ, TNF-α, IL-2, IL-12) appears to predominate and takes part in granuloma formation [75,76], the current evidence suggests that a skew to Th2 response (IL-4, IL-13) plays a significant role in the maintenance of inflammation and development of a fibrotic response in later stages [8,9,10,11,77]. Th17 response (IL-17, IL-22) arises alongside Th1 response and its absence appears to lessen inflammation and fibrosis [78,79,80].

Barrera et al. collected BAL fluid from PH patients, primed it with pigeon serum and assessed a number of cytokines. They found that patients with subacute disease showed higher percentages of CD4+ and CD8+ lymphocytes expressing IFN-γ when compared with those with the chronic form, whose CD8+ lymphocytes expressed higher IL-4 levels [9]. Similarly, culture supernatants obtained from the BAL of chronic HP patients presented higher IL-4 and lower IFN-γ(9). Studying the IL-4/IL-4R axis may also be useful to discriminate HP from sarcoidosis, as IL-4R BAL levels appear to differ, but this requires further validation [81,82]. Indeed, several studies comparing cytokine levels in the BAL of HP patients with controls and/or other ILDs have been published. 

TGF-β has been shown to have a critical role in the development of lung fibrosis and elevated levels have been demonstrated in animal models after exposure to bacteria [80,83]. Increased levels in the serum of patients with idiopathic interstitial pneumonias (IIP), including IPF, vs. healthy controls have been reported [84] and the BAL levels in HP appear to be lower when compared with IPF [85], but a complete threshold analysis has not been performed. Similar levels have been observed in patients with HP and sarcoidosis [81].

TNF-α in the BAL of patients with HP appears to be lower than on fibrotic IIP and connective tissue disease-associated ILD (CTD-ILD) [86] but not from those with sarcoidosis [81].

IL-8, a major neutrophil chemoattractant, is overexpressed in the BAL in several ILDs, including HP, sarcoidosis and IPF [85,87,88], and it demonstrated significantly different levels between the former two, even though only cases of acute HP were included [88].

Measuring the change in the BAL cytokine profile with inhalation provocation tests may also allow to distinguish HP from other ILD [89], even though the true discriminatory ability still needs to be assessed.

**Figure 1:**
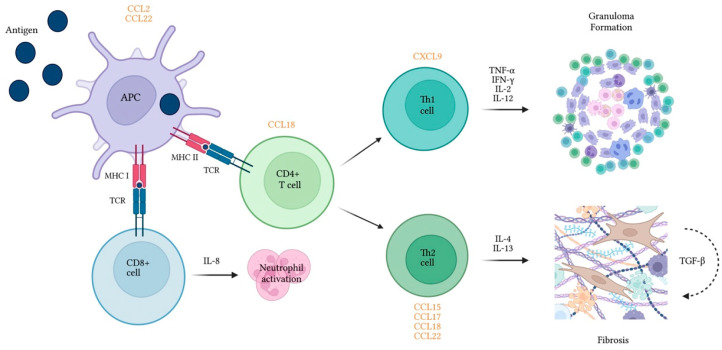
Cytokines and chemokines released by immune cells play a role in inflammatory and fibrotic processes associated with hypersensitivity pneumonitis. Chemokines are presented in orange in proximity to the cell type they recruit. APC—Antigen presenting cell; CCL—Chemokine ligand; CD—Cluster of differentiation; CXCL—Keratinocyte-derived cytokine ligand; IFN-γ—Interferon gamma; IL—interleukin; MHC—Major histocompatibility complex; TCR—T-cell receptor; TGF-β—Tumor growth factor beta; TNF-α—Tumor necrosis factor alpha.

To conclude, there is a distinctive contribution of cytokines to the inflammatory and fibrogenic processes associated with the disease (Figure 1). Differences in these profiles between HP and other ILDs and/or healthy controls have been documented, especially in the BAL, but validation for diagnostic testing is still lacking.

#### 2.1.4. Chemokines and Receptors

Chemokines are small proteins synthesized by several cell types and are recognized for their ability to stimulate cell migration. Similar to interleukins, a differential expression of chemokines has been shown in chronic HP (Figure 1) and proved useful mostly for predicting prognosis. Some studies, however, reported different levels vs. healthy controls or other ILD groups.

Chemokines such as MIP-1a/CCL3 and CCL5 are released when an APC interacts with the inciting antigen. Additionally, TH2-related chemokines (e.g., TARC/CCL17) were found in the serum of patients with HP, particularly those with an acute exacerbation [90]. Higher serum and BAL levels were also found in patients with UIP-like pattern, when compared with patients with a Nonspecific Interstitial Pneumonia (NSIP)/Organizing Pneumonia (OP) pattern [11].

CCL15 serum levels were higher in chronic HP when compared with IPF and controls [91], but discriminatory analysis remains to be performed. A study by Nukui et al. reported [92] higher CXCL9 (Th1) and CCL17 (Th2) serum levels in chronic bird-related HP compared to healthy controls [93].

The levels of CCL18 (also known as PARC and MIP-4) were initially reported to be higher in tissue biopsies of patients with HP, when compared with IPF and controls [94]. Tissue levels were inversely correlated with the extension of fibrosis and directly correlated with BAL lymphocytes, in line with the results obtained by Cai et al., who reported a correlation between BAL CCL18 and lymphocytes as well as a trend for lower CCL18 in chronic vs. acute/subacute HP. The same group found higher serum and BAL CCL18 levels in HP compared to IPF, COP and sarcoidosis [95].

Abnormal expression of CCL2 (MCP-1), an important monocyte/macrophage-mediated chemoattractant, has been documented in the lungs of patients with IPF and other ILDs [96]. Lymphocytes from patients with HP overexpress this protein in the presence of fibrocytes [97] and may display elevated BAL levels when compared with healthy controls [88,98]. Interestingly, even though serum and BAL elevations are also found in IPF, sarcoidosis and CTD-ILD [98], serum levels appear to be lower in fibrotic HP compared to IPF, with acceptable discriminative ability (AUC 74%) [99].

CCL22 (MDC) participates in macrophage recruitment and plays a pivotal role in several Th2 conditions such as asthma and eosinophilic pneumonia [100,101]. High levels in the BAL of patients with IPF and HP have been reported [85].

#### 2.1.5. Specific IgG Antibodies

Even though HP is believed to be mostly a consequence of T-cell-mediated immunity, B lymphocytes are also presented with processed antigens by innate immune cells, triggering the production of specific IgGs [8]. Resulting immune complexes appear to mediate lung injury [49,102].

Even though lists of proven and suspicious exposures and antigens are growing [8], testing for specific IgG antibodies against potential antigens plays an important role in the diagnostic approach. An elevation of these biomarkers, however, reflects previous exposure and sensitization rather than the disease itself [103]. This is corroborated by (a) the evidence of high serum levels among those regularly exposed but not diseased (b) the absence of vasculitis and neutrophilic infiltration in diseased lung tissue and (c) the absence of histologic features of HP in animal models exposed to antigens after being injected with precipitins [7,103].

There are several methods for assessing serum IgG antibodies, both qualitive and quantitative. The major limitations for their use for diagnosis are the lack of technical standardization for immunoassays and antigen preparations, cross-reactivity among antigens, variable cutoffs for quantitative assays and limited test-performance validation in specific populations [13]. Nonetheless, current clinical guidelines endorse the role of specific IgG antibodies as ancillary methods to document exposure to a culprit antigen and increase the likelihood of HP [12,13].

Jenkins et al. systematically reviewed and pooled studies on serum IgG testing using ELISA assays or precipitins and concluded that this parameter was able to discriminate HP from other ILDs with a sensitivity of 83% (95% CI, 28–98%) and a specificity of 68% (95% CI, 38–90%) [104], an suboptimal performance for it to be used as an individual diagnostic test in most clinical settings. Results from the same study revealed a capacity to distinguish patients with HP from healthy exposed subjects with a sensitivity and specificity of 90% (95% CI, 62–98%) and 91% (95% CI, 69–98%), and from healthy unexposed individuals with 93% (95% CI, 59–100%) and 100% (95% CI, 99–100%) sensitivity and specificity, respectively [104]. 

In an interesting prospective case-cohort study, Morell et al. found a positivity rate of 63% (*n* = 29) for specific IgG tests in a population of patients fulfilling the 2011 ATS/JRS/ALAT Guidelines IPF criteria, 18 of whom were confirmed to be fibrotic HP in the follow-up period [19].

Summing up, in the appropriate clinical setting and when added to other relevant sources of information, IgG testing may be useful to support the diagnosis of HP or even suggest a previously unnoticed inciting source [15]. This is particularly relevant in the case of fibrotic HP, where failure to identify an occult exposure may lead to a misdiagnosis (namely of IPF) and deprive the patient of adequate counseling for antigen avoidance and immunomodulatory therapy [105].

#### 2.1.6. Markers of Macrophage Activation

##### YKL-40 

YKL-40 (also chitinase 3-like 1) is a chitinase-like protein expressed by a variety of cells, including neutrophils, epithelial cells and APCs, in response to specific stimuli [106,107,108,109]. It plays an important role in several biological cascades in the respiratory system, inhibiting oxidant-induced injury, stimulating adaptive Th2 immunity, regulating cell apoptosis and alternative macrophage activation, ultimately contributing to fibrosis and wound healing [106]. It is not surprising, thus, that it has been described as a biomarker in diseases characterized by inflammation, fibrosis and remodeling, IPF and sarcoidosis among them [109,110,111,112]. In fact, the usefulness of YKL-40 in the diagnosis of ILD (versus healthy controls) was summarized in a systematic review and meta-analysis, which revealed overall highest serum levels in sarcoidosis when compared with other ILDs [113]. Unfortunately, studies on HP were not included. Patients with HP appear to have higher serum levels than healthy controls, with the most favorable cutoffs showing, however, suboptimal discriminatory ability [114,115,116]. On the other hand, serum YKL-40 levels seem to be lower than in other ILDs, such as idiopathic NSIP, OP and IPF [114,115]. In fact, this biomarker’s acceptable ability to discriminate between HP and IPF [114,115] is worth noting and further exploring in the future, given the clinical relevance of this issue.

#### 2.1.7. Acute Phase Reactants

##### Serum Amyloid A (SSA)

SSA is an apo-lipoprotein mainly produced in the liver by activated monocyte cells in response to proinflammatory cytokines but is also expressed by other extrahepatic cell types [117,118]. It has shown several functions, including the ability to induce the synthesis of cytokines and to attract neutrophils and mast cells.

The elevation of SSA levels has long been described in sarcoidosis [119,120,121,122], a finding substantiated by the description of the role of this molecule as an innate regulator of granulomatous inflammation in this disease [118]. Chen et al. reported the overexpression of SSA in granulomatous tissue on lung and lymph nodes of sarcoidosis patients, with a greater extent when compared to other granulomatous diseases including HP [118]. This study also showed a correlation with collagen deposition and tissue fibrosis. These findings are in line with those of higher SSA levels in patients with fibrotic vs. non-fibrotic sarcoidosis [123] and with the proposed role for SSA in ECM remodeling and fibrogenesis, favoring a profibrotic cytokine environment and metalloprotease expression by fibroblasts [124,125]. In fact, the increase in SSA levels in other fibrotic diseases has also been widely demonstrated [123,124,126,127,128]. For HP, particular emphasis should be given to the different serum levels of patients with HP vs. IPF and controls, translating into an acceptable-to-excellent discriminatory ability [126,128]. Later conflicting evidence [123], however, highlighted the need to further explore the abilities of this biomarker for the diagnosis of fibrotic HP (fHP).

**Table 1:** Studies exploring biomarkers of immune dysregulation in individuals with hypersensitivity pneumonitis.

Biomarker	Investigator	Type of Study	Population/Methods	Threshold Analysis (Cutoffs)/Other Results	Conclusion(s) of Interest	Main Limitations
BAL Lymphocytes	Patolia et al. [60]	Systematic review and meta-analysis	Studies on BAL cell counts in ILDs; comparison of BAL% lymphocytes in HP vs. IPF or sarcoidosis	No 20% BAL lymphocytes: fHP vs. IPF: Sen 69% Sp 61% fHP vs. sarcoidosis: Sen 69% Sp 26%	BAL lymphocytes: Higher in HP vs. IPF Higher in HP vs. sarcoidosis	No RCTs Possible incorporation bias of the studies reviewed cHP used as surrogate for fibrotic HP
	Adderley et al. [61]	Systematic review and meta-analysis	Studies on BAL cell analysis in ILDs; estimation of pooled BAL% lymphocytes for cHP vs. non-cHP ILDs	Yes 21.3% BAL lymphocytes: CHP vs. non-CHP ILD Sen 66.5% Sp 65.9% 21% BAL lymphocytes CHP vs. IPF/non-IPF IIP Sen 70.7% Sp 67.6%	BAL lymphocytes: Higher in cHP vs. non-cHP ILD	No RCTs Possible incorporation bias affecting the included studies Heterogeneity of cHP diagnostic criteria among studies
BAL CD4/CD8 cell ratio	Adderley et al. [61]	Systematic review and meta-analysis	Studies on BAL cell analysis in ILDs; estimation of pooled BAL ratios for CHP vs. non-cHP ILDs	No. CD4/CD8 Ratio pooled estimates: cHP 1.6 IPF 1.6 Sarc 4.6	CD4/CD8 Ratio: CHP vs. IPF not different cHP ≠ sarcoidosis	Small sample size of non-CHP groups Possible incorporation bias affecting the included studies High between-study heterogeneity
	Barrera et al. [9]	Prospective cohort	Patients with aHP, saHP, cHP and healthy controls; comparison of BAL CD4/CD8 and cytokines in BAL cell culture supernatant	No. Median CD4/CD8 Ratio: cHP 3.05 SaHP 1.3 Controls 1.3 IL-4 (pg/mL): cHP 80 saHP 25 IFN-γ (pg/mL): cHP 3.82 saHP 100 TGF-β1: No difference	CD4/CD8 Ratio: Higher in CHP vs. subacute HP and healthy controls	Former diagnostic criteria and classification of HP Only Pidgeon Breeder’s Disease included Single center study: small sample
Cytokines, Chemokines and receptors	Ye et al. [74]	Cross-sectional study	Patients with aHP, cHP and healthy controls; comparison of cytokine concentration in alveolar macrophage culture supernatant	No. IL-12 (pg/mL): cHP 15.4 Controls 1.1 IL-18 (pg/mL): aHP 201 cHP 224 Controls 56 TNF-α (pg/mL): aHP 3219 cHP 1522 controls 249	No difference between aHP and cHP	Own diagnostic and classification criteria Single center study; Small sample size
	Matěj et al. [81]	Cross-sectional study (case-control analysis)	Patients with HP and sarcoidosis; comparison of BAL cytokine concentration at diagnosis	No IL-4R (pg/mL): HP 1182 Sarc 303 PAR-2 (pg/mL): HP 2009 Sarc 330 TGF- β and TNF-α: No difference	PAR-2 as a potential tool in the differential diagnosis	Single center study; small sample HP diagnostic criteria and inciting antigens not detailed
	Sterclova et al. [82]	Cross-sectional study (case-control analysis)	Patients with cHP and sarcoidosis; determination of IL-4/IL-4R axis role	No. IL-4Ra: cHP 1190 Sarc 303 IL-4: No difference	BAL IL-4Ra significantly higher in cHP vs. sarcoidosis	Own HP diagnostic criteria Single center study; Small sample size
	Stijn et al. [85]	Cross-sectional study (case-control analysis)	Patients with cHP, IPF and controls without pulmonary disease. BAL protein comparison (multiplex) at diagnosis	No. TGF-β1 lower in cHP vs. IPF and controls IL-8 lower in cHP vs. controls MMP-8, MMP-9, MCP-1, MDC, MPO and Protein-C higher in cHP vs. controls IL-17a and IL-23, RAGE, SP-C, TIMP-1, fibronectin, eotaxin, IL-17A, IL-23, PARC, RANTES, TSLP, PlGF, FGFb, and tissue factor: no difference	No considerations on use as tools in differential diagnosis.	Multidisciplinary meeting diagnoses—criteria and inciting antigens not detailed Single center study; Small sample size
	Bruzova et al. [86]	Cross-sectional study (case-control analysis)	Patients with IPF, fIIP, CTD-ILD and cHP Comparison of BAL supernatant protein levels	No TNF-α HP 3.41 fIIP 1.52 CTD-ILD 2.33 IL4R-α and PAR2: no differences MMP-7: no differences	TNF-α higher in cHP vs. fIIP and CTD-ILD No differences in IL-4Ra, PAR-2 and MMP-7 TNF-α as a potential diagnostic tool in IIP	Inciting antigens not detailed Predominance of non-fibrotic cases in HP Single center study; relatively small sample
	Sterclova et al. [87]	Cross-sectional study (case-control analysis)	Patients with IPF and cHP. Comparison of BAL supernatant protein levels	No. I-TAC/CXCL11, IP-10/CXCL10, IL-8/ CXCL8 and ENA-78/CXCL5: no differences	No difference in the studied proteins	Former diagnostic criteria of IPF; own criteria of HP and little detailed Inciting antigens not detailed Single center study; Small sample size
	Sugiyama et al. [88]	Cross-sectional study (case-control analysis)	Patients with summer-type aHP, pulmonary sarcoidosis and control. Comparison of BAL supernatant protein levels	No. IL-8: aHP 30.8 Sarc 11.7 Controls 7.4 MCP-1: aHP 34.8 Sarc 45 Controls 10.6	IL-8 higher in HP than sarcoidosis MCP-1 higher in aHP vs. controls, not sarcoidosis	Only summer-type HP included Single center study; Small sample size
	Inoue et al. [89]	Cross-sectional study (case-control analysis)	Patients with HP and other ILDs; comparison of BAL and serum potential biomarkers after IPT with pigeon extract in avian HP and other ILDs (including non-avian HP)	No BAL G-CSF, IL-6, and IL-17 higher after IPT only in avian HP Serum leukocytes and neutrophils elevated after IPT only in avian HP	Neutrophils and the cytokine/chemokine-associated millieu increase with IPT in avian HP but changes in cytokines/chemokines should be carefully interpreted (risks of multiple comparisons)	Former diagnostic/classification criteria Single center study; small sample Only avian IPT performed
	Watanabe et al. [91]	Cross-sectional study (case-control analysis)	Patients with cHP, IPF and healthy controls; comparison of serum and BAL CC15	No Serum (μg/mL): cHP 29.1 IPF 19.7 controls19.5 BAL (μg/mL): cHP 0.76 IPF 0.54	Serum CCL15 higher in cHP vs. IPF;	Former diagnostic/classification criteria Single center study; Small sample size The assessment of inciting antigens was performed using precipitins only
	Nukui et al. [93]	Retrospective cohort (case-control analysis)	Patients with cHP and healthy volunteers; comparison of serum CXCL9, CCL17 and KL-6	No KL-6 (U/mL): cHP 1182 controls 184 CXCL9 (pg/mL) cHP 19.3 controls 10.5 CCL17 (pg/mL) cHP 543 controls 274	KL-6, CXCL9 and CCL17 higher in avian cHP vs. healthy controls	Former diagnostic criteria Only avian cHP defined only by IPT
	Cai et al. [95]	Cross-sectional study (case-control analysis)	Patients with HP and other ILDs; comparison of serum and BAL CCL18 levels	No Serum (ng/mL): HP 190 IPF 149 COP 146 Sarc 108 BAL (ng/mL) HP 13 IPF 6 RB-IL/DIP 54 COP 5 Sarc 7 iNSIP 9	CL18 level highest in HP among the investigated ILDs	Own diagnostic criteria for HP and former for IIP; saHP and cHP considered in the same group Single center study; Small sample size
	Garcia de Alba [97]	Cross-sectional study (case-control analysis)	Patients with cHP and healthy controls; comparison of serum and BAL CXCL12 levels	No Serum (pg/mL): cHP 2302 controls 813 BAL (pg/mL) cHP 493 controls undetectable	CXCL12 levels higher in cHP vs. controls. No considerations on use as diagnostic tool	Own/former HP criteria Only bird-related HP Single center study; Small sample size
	Suga et al. [98]	Cohort study (case-control analysis)	Patients with ILD (IPF, AIP, CTD-ILD, OP and HP); Comparison of serum and BAL MCP-1/CCL2 levels	No BAL levels higher in IPF vs. HP Serum levels higher in IPF, Sarc, and CTD-ILD vs. HP and controls	Differences in the pattern of MCP-1 in BALF and serum may help in the differential diagnosis of ILD.	Former diagnostic criteria Single center study; Small sample size
Specific IgG antibodies	Jenkins et al. [104]	Systematic Review and meta-analysis	Studies on serum IgG testing and questionnaires in HP	No. HP vs. other ILDs Sen 83% Sp 68% HP vs. E-controls Sen 90% Sp 91% HP vs. u-controls Sen 93% Sp 100%	IgG testing insufficient to distinguish HP from other ILDs but can be useful in screening for exposures and provide supportive of HP	No RCTs Small studies included Risk of incorporation bias
Chitinase-3-like protein 1 (YKL-40)	Long et al. [114]	Retrospective cohort (case-control analysis)	Patients with HP, IPF, iNSIP, COP and healthy controls; Comparison of serum and BAL YKL-40	Yes Serum (ng/mL) HP 127 IPF 214 iNSIP 184 COP 213 controls 39 a/saHP 179 cHP 117 BAL (ng/mL) HP 21 IPF 9 a/saHP 42 cHP 15 control 3 Optimal cutoff serum: HP vs. controls: 47 AUC 0.90 HP vs. IPF: 134 AUC 0.727	Serum YKL-40 higher in HP vs. controls but lower vs. other ILDs BAL YKL-40 higher in HP vs. controls and IPF No considerations on use as diagnostic tool	Former diagnostic and classification criteria Single center study; Small sample size
	Sanchez-Diaz et al. [115]	Cross-sectional study (case-control analysis)	Patients with HP, IPF, sarcoidosis and healthy controls; Comparison of serum YKL-40 and KL-6	Yes Serum YKL-40 HP 56 ng/mL IPF NM Optimal cutoff for HP vs. IPF: Serum YKL-40 121 ng/mL AUC 0.741 Serum KL-60: 1441 U/mL AUC 0.702	Serum HP higher in HP vs. IPF	Ancient diagnostic criteria; own classification criteria Single center study; Small sample size
Serum Amyloid A	Vietri et al. [127]	Prospective cohort (case-control analysis)	Patients with IPF and non-IPF ILDs including sarcoidosis, cHP, PLC and healthy controls; comparison of SAA concentrations in the different groups	No SAA: IPF6418 ng/mL; cHP 4494 ng/mL Optimal cutoff for IPF vs. cHP: 5397 ng/mL AUC 0.79	SAA Higher in IP levels vs. other ILDs; potential to differentiate IPF from cHP	Diagnostic criteria of diseases not detailed; inciting antigens not detailed Single center study; Small sample size
	Bergantini et al. [128]	Coss-sectional study (case-control analysis)	Patients with IPF, sarcoidosis, cH. Comparison of fibrotic/inflammatory markers	Yes. KL-6 (U/mL) cHP 1146 Sarc 537 IPF 2062 SAA (ng/mL) cHP 4022 Sarc 4370 IPF 7031 Optimal cutoff for cHP vs. IPF: KL-6: 2206 AUC 0.74 SAA: 53,971 AUC 0.85 KL-6 + SAA: AUC 0.79	KL-5 different in cHP vs. IPF and sarcoidosis SAA different in cHP vs. IPF Combined panel might improve diagnostic accuracy in multidisciplinary setting	Diagnostic and classification criteria not detailed Single center study; Small sample size

Studies comparing biomarkers of immune dysregulation in human individuals with HP vs. other ILDs and/or healthy controls. Biomarkers of other classes are highlighted. aHP—Acute hypersensitivity pneumonitis; AIP—Acute interstitial pneumonia cHP—Chronic Hypersensitivity Pneumonitis; COP—Cryptogenic Organizing Pneumonia CTD—Connective Tissue Disease; fIIP—Fibrosing Idiopathic Interstitial Pneumonia; ILD—Interstitial Lung Disease; iNSIP—idiopathic nonspecific interstitial pneumonia IPF—Idiopathic Pulmonary Fibrosis; IPT—inhalation provocation testing; PLCH—Pulmonary Langerhans Cell Histiocytosis; RCT—Randomized Controlled Trial; SAA—Serum Amuloid A; saHP—subacute hypersensitivity pneumonitis; Sarc—Sarcoidosis; Sen -Sensitivity; Sp—Specificity.

### 2.2. Epithelial Cell Dysfunction

**Figure 2:**
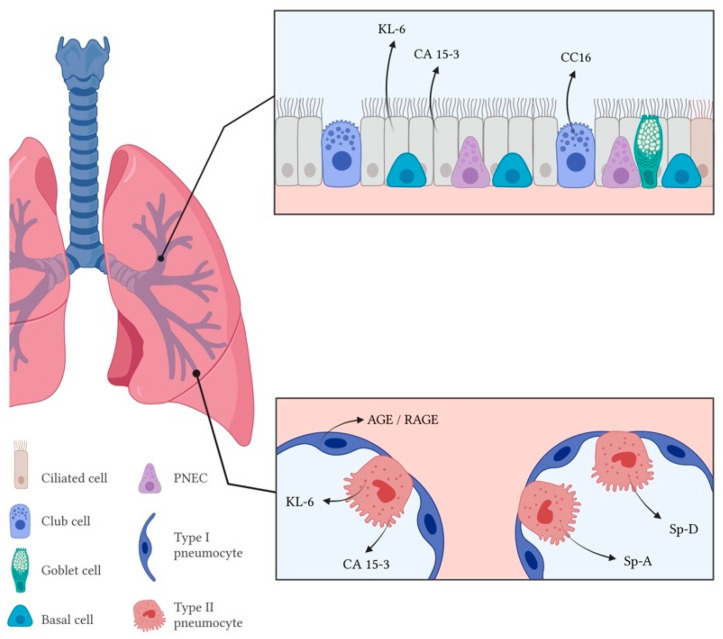
Markers of epithelial cell dysfunction of respiratory epithelium in the airway (upper rectangle) and alveolar space (lower rectangle) in hypersensitivity pneumonitis. PNEC—Pulmonary neuroendocrine cells; KL-6—Krebs von den Lungen-6; CA 15-3—Carcinoma antigen 15-3; CC16—Club-cell protein 16; RAGE—Receptor for advanced glycation end products; Sp-A—Surfactant protein A; Sp-D—Surfactant protein D.

Lung epithelial cells have a crucial role in defending the host from external aggressions through different mechanisms, including secretion of mucus, surfactant and several other defense proteins [129]. Epithelial cell injury and apoptosis are well documented in hypersensitivity pneumonitis [130], as well as significant changes in levels of epithelial cell-related biomarkers (Figure 2, Table 2).

#### 2.2.1. Mucin-Associated Antigens

##### Krebs Von Den Lungen-6 (KL-6)

KL-6, a sub-molecule of Mucin 1, is a high molecular weight glycoprotein expressed by type 2 pneumocytes and bronchiolar epithelial cells and has been one of the first biomarkers of lung damage to be reported, both in the serum and the BAL [41,131,132]. Correlation with bronchiolar epithelial cell damage and regeneration and with progressive interstitial thickening boosted it as a promising biomarker for detecting, assessing activity and predicting prognosis of several ILDs [133,134]. 

Evidence has consolidated the utility of serum levels in differentiating patients with ILD, including HP, from subjects without ILD [41,93,135,136,137,138], but low diagnostic accuracy limits the utility in ILD differential diagnosis [133,139,140]. 

In an early study, Takahashi et al. described significantly higher serum KL-6 concentrations in patients with farmer’s lung disease when compared with farmers without the disease (including the same subjects prior to developing the disease) [137]. Interestingly, lower diffusing capacity for carbon monoxide (DLCO) and KCO levels were also noted in farmers with high serum KL-6 levels, regardless of fulfillment of the criteria for HP [137]. Ji et al. later found higher serum KL-6 levels in bird fanciers vs. controls, and in acutely symptomatic fanciers vs. asymptomatic ones [141]. In this study, KL-6 was also associated with specific IgG antibodies and this correlation was stronger in symptomatic subjects.

Okamoto et al. reported an increased serum value of KL-6 in chronic HP when compared with IPF, CTD-ILD and sarcoidosis and were able to determine an optimal cutoff value of 1115 U/mL for the differential diagnosis with IPF, noting low specificity as a major limitation [142]. Onishi et al. showed significantly higher serum levels of KL-6 in cHP patients when compared with IPF [143]. 

Besides the study from Lanzarone et al., who found a correlation between KL-6 levels and ground glass opacities score [140], we found no other studies on KL-6 discriminating fHP, but there is some evidence on the correlation between KL-6 levels and fibrotic extension in other entities [144,145]. These data point to a plausible role of KL-6 in identifying individuals with fibrotic HP among healthy subjects and as a surrogate marker for antibody titration in individuals whose exposure cannot be identified.

##### CA 15-3 and CA125

Like KL-6, Carcinoma Antigen 15-3 (CA 15-3) assays target a Mucin 1 (MUC1)-derived glycoprotein and are believed to reflect fibrotic processes and immunologic disease activity in several ILDs [146,147]. Compared with KL-6, this test has the advantages of wider implementation and lower cost associated with its use in breast cancer. CA 15-3 levels were found to correlate with KL-6 levels in patients with fibrotic ILDs, including HP, and to correlate with fibrosis scores [147,148,149,150]. The diagnostic ability of the test has not, however, been explored, to the best of our knowledge, but it provides a potential field for future research.

#### 2.2.2. Surfactant-Associated Proteins

Surfactant protein A (Sp-A) and surfactant protein D (Sp-D) are members of the epithelial collectins, a group of soluble pattern-recognition receptors produced by type II pneumocytes and involved in the innate immune response in both pulmonary and non-pulmonary tissues [151,152,153]. They are major protein components of lung surfactant, being responsible for its homeostasis [151,152,153]. Alongside KL-6, Sp-D is one of the most widely accepted diagnostic tests for ILD, particularly in Japan, being useful as a marker of disease activity as well [142]. Tanaka et al. first described the elevation of serum Sp-D levels in two patients with mushroom worker’s lung, reporting a decrease with antigen avoidance and steroid therapy [154]. 

Janssen et al. demonstrated a relevant discriminatory capacity of serum Sp-D in patients with bird fancier’s lung vs. controls (AUC 0.96) [155]. 

Okamoto et al. analyzed serum Sp-D levels in acute and chronic HP and compared them with three other groups of patients diagnosed with IPF, sarcoidosis and CTD-ILD. The authors report higher Sp-D levels in acute vs. chronic HP, and in both when compared with every other group. Additionally, at an optimal cutoff of 209 ng/mL, this biomarker could reasonably discriminate between chronic HP and IPF (AUC = 0.729) [142]. Similarly, Onishi et al. also demonstrated higher levels of Sp-D in chronic summer-type hypersensitivity pneumonitis when compared with IPF [143]. 

Guzman et al. first reported the increase in Sp-A+ alveolar macrophages in HP [156]. This was later corroborated by other groups, who reported significant increases in Sp-A in the BAL of patients with HP vs. healthy controls [157,158,159]. Hamm et al. and Phelps et al. reported increased levels in other ILDs, namely sarcoidosis and IPF, but did not find differences from patients with HP [157,159].

This evidence underlines the potential utility of surfactant proteins, particularly Sp-D, in the diagnosis of HP, although both still require proper validation for clinical use.

#### 2.2.3. Club-Cell Protein

Club-Cell Protein 16 (CC16, also known as CC10 and uteroglobin) is secreted primarily by the Club Cells (non-ciliated bronchial epithelial secretory cells). Its functions are not completely understood, but it appears to prevent fibrosis in animal models, possibly by reducing profibrotic cytokine and metalloproteinase activity [160,161,162]. On the other hand, cytokines such as IFN-γ, TNF-α and IL-10 have proven capable of increasing CC16 expression in different cell populations [163,164,165].

The dysregulation of CC16 homeostasis has been associated with several airway and parenchymal diseases [166]. Concerning ILDs, elevated levels are found on IPF, CTD-ILD (including those associated with Sjogren’s, rheumatoid arthritis and systemic sclerosis) and HP [167,168,169,170]. Elevated serum levels in HP when compared with healthy controls [170] are relevant but unlikely sufficient to reach clinical practice. On the other hand, even though ROC analysis describes a sub-optimal discriminatory power, lower serum levels (in conjunction in CTD-ILD) vs. IPF [169] could prove useful in daily clinical practice but further studies on this matter are required.

#### 2.2.4. AGE and RAGE

The receptor for advanced glycation end products (RAGE) is a member of the immunoglobulin superfamily, expressed mainly in the basal membrane of type 1 pneumocytes. It was first identified as the receptor for advanced glycation end products but also binds to a large variety of endogenous ligands, including ECM components such as collagen type 1 and type 4 [171]. Several effects have been attributed to this molecule. After binding, RAGE initiates intracellular cascades that lead to the transcription of proinflammatory genes [172]. It was also shown to promote leukocyte adhesion and recruitment by binding macrophage integrin MAC-1 [173] and inducing the expression of vascular cell adhesion molecule (V-CAM) in endothelial and mesothelial cells [174,175]. Finally, it is also involved in type 1 pneumocyte differentiation, epithelial adhesion to the basement membrane and lung re-epithelialization [176,177,178].

AGEs result from the reaction between monosaccharides and free amino protein groups [179]. Besides being related with oxidative stress, aging and inflammation, they have also been shown to act on ECM protein cross-links and reduce tissue viscoelasticity [180,181].

Even though distinct patterns of expression, particularly for RAGEs, have been found in different models of lung fibrosis, decreased RAGE and increased AGE expression have been described in the lungs and serum of patients with IPF when compared with controls [182,183,184], which is not completely corroborated by other studies [85]. The AGE/soluble RAGE (sRAGE) ratio was found to be different between IPF and cHP, with moderate discriminatory ability [183]. Interestingly, both serum AGE, sRAGE and AGE/sRAGE ratio distinguished patients’ cHP from fibrotic NSIP [183]. The usefulness of these biomarkers in the differential diagnosis of fibrotic HP deserves further exploring.

**Table 2:** Studies exploring biomarkers of epithelial cell dysfunction in individuals with hypersensitivity pneumonitis.

Biomarker	Investigator	Type of Study	Population(s)/Methods	Threshold Analysis (Cutoffs)/Other Results	Conclusion(s) of Interest	Main Limitations
Krebs von der Lungen (KL-6) and surfactant proteins (SPs)	Takahashi et al. [137]	Cross-sectional study (case-control analysis)	Dairy farmers with and without HP (controls); comparison of serum KL-6 levels in HP, precipitin positive (Ab+) and precipitin negative (Ab−) controls	No KL-6 (U/mL): HP 1263 Ab+ controls: 328; Ab− controls: 207	KL-6 higher in farmers with HP vs. controls and higher in seropositive vs. seronegative controls KL-6 higher in individuals after diagnosis vs. before diagnosis	Only farmers’ lung disease; Single center study; Small sample size
	Okamoto et al. [142]	Retrospective longitudinal study (case-control analysis)	Patients with cHP, aHP, IPF, CVD-ILD; Comparison and validation of serum KL-6 and SP-D	Yes KL-6 and SP-D higher in aHP and cHP vs. IPD, CVD-ILD and sarcoidosis Not different in aHP vs. cHP Optimal cutoff for cHP vs. IPF: KL-6 1115 U/mL AUC 0.771 SP-D 209 ng/mL AUC 0.729 KL-6 and SP-D lower after 1-month steroid therapy	Higher KL-6 and SP-D shoul raise suspicion of HP Potential use in disease management	Own HP diagnostic criteria of HP; Former diagnostic criteria of IPF and sarcoidosis; Single center study.
	Onishi et al. [143]	Cross-sectional study (case-control analysis)	Patients with cHP and IPF; comparison of serum biomarkers	No KL-6: cHP 1506 U/mL IPF 914 U7 mL SPD cHP 235 ng/mL IPF 156 ng/mL	KL-6 and SP-D higher in cHP vs. IPF Both biomarkers included in proposed composite criteria for chronic summer-type HP	Only summer-type HP Former diagnostic/classification criteria Single center study; small sample
	Janssen et al. [155]	Retrospective longitudinal study (case-control analysis)	Patients with HP and healthy exposed and unexposed controls; comparison and validation of serum KL-6 and SP-D	Yes KL-6: cHP 883 U/mL E-controls 371 U/mL U-controls 177 U/mL SP-D: cHP 201 ng/mL U-controls 68 ng/mL Optimal cutoff: KL-6 275 U/mL AUC 0.98 SP-D 98 ng/mL AUC 0.96	KL-6 and SP-D decrease with antigen avoidance; potential markers of disease	Criteria for diagnosis and classification of HP not detailed Only bird fancers’ lung disease Single center study; Small sample size
	Hamm et al. [157]	Cross-sectional study (case-control analysis)	Patients with HP, sarcoidosis and controls without ILD; comparsion of BAL supernatant SP-A levels	No BAL SpA: HP 9 μg/mL Sarc 8 μg/mL Controls 4.0μg/mL	BAL SP-A are elevated in sarcoidosis and HP but not specific or diagnostic	Own diagnostic criteria for HP and sarcoidosis Single center study; Small sample size
	Phelps et al. [159]	Cross-sectional study (case-control analysis)	Patients with IPF, HP and controls; comparison of SP-A	No BAL SP-A: HP 13.11 ug/mL IPF 7.99 μg/mL controls 4.77 μg/mL	BAL SP-A elevated in IPF (both vs. HP and controls); significance of the finding incompletely understood	IPF diagnostic criteria not detailed; Own HP criteria Only avian antigen-induced HP Single center study; Small sample size
Club-cell protein (CC16)	Buendia-Roldan et al. [169]	Cross-sectional study (case-control analysis)	Patients with IPF, non-IPF ILD (CTD-ILD + cHP) and healthy controls; comparison and validation of serum and BAL CC16	Yes. Serum CC16 higher in non-IPF vs. controls Optimal cutoff for IPF vs. non-IPF: 41 ng/mL; AUC 0.68	High CC16 levels may increase suspicion of IPF and may complement other findings	Outdated diagnostic criteria for IPF and HP Time of blood sample collection not mentioned/Patient treatment at the time not detailed Single center study; Small sample size
	Barnes et al. [170]	Case-control study	Patients with HP and age-matched controls; comparison of selected serum biomarkers (secondary outcome)	No CC16 HP: 36.3 ng/m controls 15.0 ng/mL	CC16 associated with increased odds of HP	HP diagnosis based on administrative data/partly on clinical unmentioned criteria Patient treatment at the time of sample collectionnot detailed
Advanced glycation end products/Receptor for Advanced glycation end products (AGE/RAGE)	Machahua er al. [183]	Prospective cohort (case-control analysis)	Patients with IPF, cHP, fNSIP and controls; Comparison and validation of serum AGEs, sRAGE and AGEs/sRAGE	Yes Optimal cutoff for cHP vs. NSIP: AGEs 19.25 ug/mL (AUC 0.883); sRAGE 782.6 pg/mL (AUC 0.887); AGE/sRAGE 25.7‰ (AUC 0.882) Optimal cutoff for cHP vs. IPF: AGEs and sRAGE: NM (low AUC), AGE/sRAGE NM (AUC = 0.713)	AGE higher in cHP vs. controls; sRAGE lower in cHP vs. fNSIP and vs. controls IPF vs. cHP: AGE and sRAge not different; AGE/sRAGE as potential diagnostic tools in fibrosing ILDs	Outdated diagnostic criteria for IPF and HP Patient treatment at the time not detailed Single center study; Small sample size

Studies comparing biomarkers of epithelial cell dysfunction in human individuals with HP vs. other conditions and/or healthy controls. BAL—bronchoalveolar lavage; cHP—chronic hypersensitivity pneumonitis; CVD-ILD—Collagen vascular disease-related interstitial lung disease; IPF—idiopathic pulmonary fibrosis fNSIP—fibrotic non-specific interstitial pneumonitis; NM: not mentioned; sarc—sarcoidosis.

### 2.3. Fibrogenesis and ECM Remodeling

Fibroblast recruitment and activation in the context of imbalanced regulatory cytokine milieu culminate in excessive production and deposition of extracellular matrix (ECM) [130]. These pathogenic processes have also been active areas of research, including on the potential use of the associated molecules as diagnostic tools in ILD, including HP (Table 3).

#### Matrix Metalloproteinases (MMPs)

Matrix metalloproteinases (also known as matrixins) are zinc-dependent endoproteases that have long been considered as the main effectors of ECM degradation, modulating the activity of ECM mediators and the behavior of smooth muscle, epithelial and immune cells [185,186]. MMPs were believed to prevent fibrotic scarring, which was understood as a dysbalance between deposition and degradation of ECM components. However, the finding of increased activity both in repair/remodeling processes and in complex fibrotic conditions as IPF challenged this concept [185,186]. Even though many of the 23 members of the MMP family play different roles in the pathogenesis of lug fibrosis, only a few have been studied as diagnostic biomarkers in ILD, mostly in IPF. MMP-1, MMP-7, MMP-3, MMP-8 and MMP-9 are overexpressed, and the combination of MMP-7 and MMP-1 appears to accurately differentiate IPF from healthy controls [85,187]. This finding was corroborated by Morais et al., who found an interesting ability for both MMP-7 and MMP-1 to discriminate IPF from other ILDs, including fibrotic HP [188]. Bruzova et al. and Maldonado et al., on the other hand, did not find a difference on the levels of MMP-7 between IPF and other fibrotic IIPs [86,189]. The latter, however, described an acceptable ability of MMP-28 to distinguish IPF from non-IPF fibrosis but not fibrotic HP from fibrotic autoimmune ILD [189]. Interestingly, in the same paper, levels of this enzyme were higher in IPF-UIP compared to non-IPF-UIP, suggesting that different mechanisms result in the expression of different molecules, even though the radiological and morphologic outcomes are similar.

Little is further known about the role of MMPs in the diagnosis of fibrotic HP. A deeper knowledge of patients’ serum and BAL levels might not only expand the short list of validated diagnostic biomarkers but also the knowledge on the common and distinct pathways of ECM deposition in fibrotic HP and other fibrotic ILDs.

**Table 3:** Studies exploring biomarkers of fibrogenesis/ECM remodeling and from other sources in individuals with hypersensitivity pneumonitis.

Biomarker	Investigator	Type of Study	Population(s)/Methods	Threshold Analysis (Cutoffs)/Other Results	Conclusion(s) of Interest	Main Limitations
Matrix metalloproteinases (MMPs)	Rosas et al. [187]	Cross-sectional study (case-control analysis)	Patients with IPF, saHP/cHP, Sarcoidosis, COPD; Comparison of serum protein concentration (multiplex assay)	Yes. MMP7 and MMP1 higher in IPF vs. HP (2.3 and 1.31-fold, respectively) Optimal cutoffs and AUC: not mentioned Combination of high MMP1 + High MMP7: S 96.3% Sp 87.2%	Serum MMP1 and MMP7 as potential biomarkers in the differential diagnosis of IPF and HP	Outdated diagnostic criteria for IPF and HP Only avian inciting antigens/exposure Time of sample collection not mentioned/Patient treatment at the time not detailed Single center study; Small sample size
	Morais et al. [188]	Cross-sectional study (case-control analysis)	Patients with IPF and non-IPF ILD (including HP); Comparison of serum MMP-7 and MMP-1	Yes (IPF vs. other ILDs) MMP-1 higher in IPF vs. non-IPF-UIP Optimal cutoff for IPF vs. ther ILDs: MMP-1: 4.15 ng/mL AUC 0.63 MMP-7: 3.91 ng/mL, AUC 0.73 Combination: AUC 0.74	Potential role of serum MMP-1 and MMP-7 as diagnostic biomarkers in IPF	HP not as independent group; Former diagnostic criteria of IPF and HP Single center study; Small sample size
	Maldonado et al. [189]	Cross-sectional study (case-control analysis)	Patients with IPF and non-IPF fibrosis (including fHP) and healthy controls; Comparison and validation of MMP28 concentration in two cohorts	Yes. MMP28 higher in IPF vs. non-IPF and controls; Optimal cutoff for IPF vs. non-IPF: 4.5 ng/mL AUC 0.72 and 0.69	MMP28 as new biomarker ofr differential diagnosis of IPF with cHP and fibrotic autoimmune driven-ILD	HP not as independent group and diagnostic criteria not detailed
Lipid mediators/Adipokines	D’Alessandro et al. [99]	Cross-sectional study (case-control analysis)	Patients with IPF and fHP; comparison and validation of BAL and serum multiplex lipid profiling	Yes. Optimal cutoff BAL: Apo A1 20.99 ng/mL; Apo C3 3.62 ng/mL Apo C3 3.62 ng/mL. Combined performance: AUC 81% Optimal cutoff serum: Apo A1 12.0 ng/mL; CCL2 0.88 ng/mL Apo C3 11.53 ng/mL. Combined model performance: AUC 93%	BAL Apo A1, adipsin, Apo C3 and APN higher in HP vs. IPF Serum Apo A1 higher in HP; MCP-1 (CCL2) and Apo C3 lower in HP vs. IPF Overall performance better in BAL vs. serum	Diagnostic criteria of fHP not detailed Single center study; Small sample size

Studies comparing biomarkers of fibrogenesis/ECM remodeling and from other sources in human individuals with HP vs. other ILDs/healthy controls. Biomarkers of other classes are highlighted. APN—adiponectin; AUC—area under curve; BAL—bronchoalveolar lavage cHP—chronic hypersensitivity pneumonitis; COPD—chronic obstructive pulmonary disease IPF—idiopathic pulmonary fibrosis; MCP-1/CCL2 Monocyte Chemoattractant Protein-1/ Chemokine (CC-motif) ligand 2; saHP—subacute hypersensitivity pneumonitis.

### 2.4. Other Sources

#### 2.4.1. Metabolic Biomarkers

##### Lipid Mediators

Apo A1 is the major component of high-density lipoprotein particles and has shown anti-inflammatory properties in models of lung injury [190,191].

Decreased levels of Apo A1 have been described after high-throughput, whole-proteome BAL analysis of patients with IPF [192]. Compared to fHP, D’Alessandro et al. recently reported significantly lower levels of this molecule in both the serum and the BAL, with an AUC of 93% for the latter [99]. This study also reports a reasonable ability of Apo C3, another lipoprotein, to discriminate IPF from fHP (AUC of 69% and 68%, respectively, for measurements in the serum and in the BAL).

##### Adipokines

Adipose tissue’s secretory function has been increasingly recognized. Changes to the homeostasis of adipokines in patients with ILD have been documented, including exacerbations [193,194] and these proteins appear to regulate biological activities in endothelial, fibroblast and immune cells in several tissues [195]. D’Alessandro et al. showed distinct patterns of expression of adipsin and adiponectin in the BAL of patients with IPF and fHP and created a discriminatory model using these biomarkers [99].

### 2.5. Future Directions

Novel categories of biomarkers (e.g., exosomes, mitochondrial DNA, microRNA, quantitative imaging, transcriptomics, microbiome related) are emerging and may one day help clinicians establish or rule out the diagnosis of HP with confidence. Moreover, high-throughput processes and analysis of the existing and upcoming data may significantly enhance biomarker research in HP, as they have in other entities [196].

The significance of biomarkers in ILD is still distant from oncology, but the achievements in this area may serve as an example of the potential that molecular research holds. Even though we are still probably far from guiding therapy according to specific molecular biomarkers in ILD, their inclusion in the protocols of most clinical trials could be the first steps in the journey toward daily clinical practice. Moreover, drugs targeting molecules in the fibrotic mechanistic pathway are already a reality [197,198] and research in new compounds affecting these pathways keeps evolving and is on the verge of providing alternative compounds (Table 4). 

## 3. Discussion

Fibrotic HP is associated with significant morbidity and mortality and its phenotype resembles that of other ILDs, with IPF at the forefront [19]. In most fibrotic ILDs, predicting progression is one of the most relevant issues for the clinician, as lung function decline is associated with increased mortality [201]. However, this should not be the only concern, particularly in the case of HP, where the relationship between early diagnosis, the detection of the culprit antigen and patient outcomes [21] highlight the relevance of establishing a confident diagnosis.

Biomarkers are objectively measured indicators of biologic processes and play a major role in the diagnosis and management of a wide range of diseases in different organs and systems [202]. With regard to the lung, measuring molecule concentrations and/or cell constituents in BAL fluid has the advantage of portraying more closely the pathophysiology of the organ when compared with serum sampling. However, the development and validation of useful diagnostic biomarkers has proceeded at a slow pace [44]. Even though several biomarkers have demonstrated accuracy in patients with ILD vs. individuals without ILD, this is of limited clinical relevance [44]. On the other hand, finding a marker accurately able to discriminate between fHP and different fibrotic ILDs is of the highest clinical relevance [13], but this has been limited by the overlapping mechanisms of inflammation, airway and parenchymal damage and fibrogenesis between diseases. In the case of HP, understanding its complex pathogenesis, including heterogeneous mechanisms of sensitization, the myriad of inciting antigens and diverse clinical presentations is crucial to consensually define the disease, distinguish it from other ILDs and consequently develop research [8,12], namely in diagnostic biomarkers.

We found a relatively large number of serum and BAL biomarkers that were able to discriminate between patients with HP and individuals without ILD and, less frequently, with other ILDs. However, we found several issues limiting support for clinical application of diagnostic biomarkers in fibrotic HP. These include almost invariably small sampling and, very importantly, heterogeneity in diagnostic (gold-standard) criteria across the studies, which has been aggravated by the recent changes to the classification of the disease. There is no evidence from clinical trials, lack of data on cost-benefit analysis [203] and validation with threshold/accuracy analysis has been performed only in a minority of studies. Thus, the existing evidence was overall deemed insufficient to justify the incorporation of the reviewed biomarkers into current clinical guidelines and daily practice, with some exceptions. BAL lymphocyte counts and specific serum IgGs are, perhaps, the two more relevant ones, and their input is widely considered in the context of multidisciplinary discussion, even though their performance appears lower than desirable [12] and their clinical utility is repeatedly a subject of discussion. Larger prospective, multicentric studies using current criteria for fibrotic and non-fibrotic HP would probably enlighten their performance in the current practice and perhaps increase the confidence In their role in establishing the diagnosis in the multidisciplinary setting. On the other hand, even though biomarkers such as KL-6, SP-D, YKL-40 and apolipoproteins have not made their way to the day-to-day diagnostic approach to fibrotic HP in most tertiary centers, they have shown, in our opinion, promising results on research contexts and have the potential to translate into clinical practice once reproducibility is further confirmed.

## 4. Conclusions

Even though the development and validation of biomarkers are of particular interest and may be crucial to the simplicity of diagnosing and treating interstitial lung disease, they have been running at a slower pace than desired. As we demonstrated, the journey in the search for diagnostic biomarkers in fibrotic HP has been hindered by the delay in reaching consensus on diagnostic criteria, by heterogeneous classification of the disease and by the conduction of studies with small samples. With current evidence limiting clinical application, the future may lie in the application of combined panels of existing molecules or in the development of emerging biomarker categories. Still, the key to success could simply be in validating the existing ones, alone or in combination, in prospective, multicentric and well-characterized cohorts.

## Figures and Tables

**Table 4:** Drugs with potential use in fibrotic HP undergoing clinical trial and their effect in previously mentioned biomarkers.

Name	Class	Mechanism of Action	Target Disease(s)	Biomarker Repercussion	Study Phase
BI 1015550	Anti-inflammatory, antifibrotic	Preferential PDE 4B inhibitor	IPF PPF-ILD	Inhibition of TNF-α and IL-2 release [199]	Phase III Clinical Trials (recruiting) NCT05321069 NCT05321082
BMS-986278	Antifibrotic	LPAR-1 antagonist	IPF PPF-ILD	Potential reduction in serum ECM-neoepitope biomarkers [200]	Phase II Clinical Trial (active) NCT04308681

## Data Availability

No new data were created or analyzed in this study. Data sharing is not applicable to this article.
